# COVID-19-Associated Pulmonary Aspergillosis in Russia

**DOI:** 10.3390/jof7121059

**Published:** 2021-12-10

**Authors:** Olga Shadrivova, Denis Gusev, Maria Vashukova, Dmitriy Lobzin, Vitaliy Gusarov, Mikhail Zamyatin, Anatoliy Zavrazhnov, Mikhail Mitichkin, Yulia Borzova, Olga Kozlova, Ekaterina Desyatik, Ekaterina Burygina, Svetlana Ignatyeva, Ellina Oganesyan, Natalya Vasilyeva, Nikolay Klimko, Working Group

**Affiliations:** 1 North-Western State Medical University Named after I.I. Mechnikov, 191015 Saint-Petersburg, Russia; olshadr@mail.ru (O.S.); borzova-y@mail.ru (Y.B.); olgakozlova07@gmail.com (O.K.); e_desyatik@mail.ru (E.D.); katerina.prokura@gmail.com (E.B.); ign0452@mail.ru (S.I.); Ellina.Oganesyan@gmail.com (E.O.); natalya.vasileva@szgmu.ru (N.V.); 2 Botkin’s Hospital, 195067 Saint-Petersburg, Russia; gusevden-70@mail.ru (D.G.); mavashukova@yahoo.com (M.V.); 3 Military Medical Academy Named after S. M. Kirov, 194044 Saint-Petersburg, Russia; dlobzin89@mail.ru; 4 Pirogov National Medical and Surgical Center, 105203 Moscow, Russia; gusarovvg@pirogov-center.ru (V.G.); mnz1@yandex.ru (M.Z.); 5 City Mariinskaya Hospital, 191014 Saint-Petersburg, Russia; zaa.70@mail.ru (A.Z.); meg657030@mail.ru (M.M.)

**Keywords:** invasive aspergillosis, *Aspergillus* spp., COVID-19, COVID-19-associated pulmonary aspergillosis, CAPA

## Abstract

We studied the risk factors, etiology, clinical features and the effectiveness of therapy of COVID-19-associated pulmonary aspergillosis (CAPA) in adult patients. In this retrospective study, we included 45 patients with proven (7%) and probable (93%) CAPA. The ECMM/ISHAM, 2020 criteria were used to diagnose CAPA. A case-control study was conducted to study the risk factors of CAPA; the control group included 90 adult COVID-19 patients without IA. In CAPA patients, the main underlying diseases were diabetes mellitus (33%), and hematological and oncological diseases (31%). The probability of CAPA developing significantly increased with lymphocytopenia >10 days (OR = 8.156 (3.056–21.771), *p* = 0.001), decompensated diabetes mellitus (29% vs. 7%, (OR = 5.688 (1.991–16.246), *p* = 0.001)), use of glucocorticosteroids (GCS) in prednisolone-equivalent dose > 60 mg/day (OR = 4.493 (1.896–10.647), *p* = 0.001) and monoclonal antibodies to IL-1ß and IL-6 (OR = 2.880 (1.272–6.518), *p* = 0.01). The main area of localization of CAPA was the lungs (100%). The clinical features of CAPA were fever (98% vs. 85%, *p* = 0.007), cough (89% vs. 72%, *p* = 0.002) and hemoptysis (36% vs. 3%, *p* = 0.0001). Overall, 71% of patients were in intensive care units (ICU) (median—15.5 (5–60) days), mechanical ventilation was used in 52% of cases, and acute respiratory distress syndrome (ARDS) occurred at a rate of 31%. The lung CT scan features of CAPA were bilateral (93%) lung tissue consolidation (89% vs. 59%, *p* = 0.004) and destruction (47% vs. 1%, *p* = 0.00001), and hydrothorax (26% vs. 11%, *p* = 0.03). The main pathogens were *A. fumigatus* (44%) and *A. niger* (31%). The overall survival rate after 12 weeks was 47.2%.

## 1. Introduction

Invasive aspergillosis (IA) is a well-known complication of intensive cytostatic and immunosuppressive therapy, organ and tissue transplantation. However, at present, the risk group for developing IA has significantly expanded to encompass other categories of patients, including patients in intensive care units (ICUs) with severe influenza and COVID-19 [1,2]. Studies showed a high incidence of IA in patients with COVID-19 treated in the ICU, especially with mechanical ventilation. The frequency of IA in intubated patients may exceed 30% [3,4,5,6]. Taking into account that severe respiratory distress requiring intensive care develops in 5–20% of hospitalized patients with COVID-19, the problem of COVID-associated invasive aspergillosis (CAPA) is becoming increasingly urgent [7,8,9,10].

We were the first to analyze the data of five patients with COVID-19 and laboratory-confirmed IA in Russia [11].

The aim: to study the risk factors, etiology and clinical features of CAPA, and the effectiveness of therapy in adult patients.

## 2. Materials and Methods

In a retrospective study (June 2020–August 2021), we included 45 patients with proven (7%) and probable (93%) CAPA. The main group consisted of 45 adult COVID-19 patients, 69% of whom were male, and the median age was 62 years (range 34–82). These patients accounted for 5.6% (45/803) of all adult IA patients from our registry (1998–2021). A case-control study (one patient of the main group per two patients of the control group) was conducted to study the features of CAPA and the risk factors for its development. The control group included 90 adult COVID-19 patients without IA, similar in demographic characteristics and background conditions. The median age of patients in the control group was 63 years (25–82), with men constituting 67%.

The risk factors, etiology of IA, clinical features of the disease course and results of CAPA treatment were analyzed in addition to demographic and anamnestic data of patients with COVID-19. ECMM/ISHAM 2020 criteria were used for the diagnosis of CAPA, and EORTC/MSGERC 2020 criteria were used for the evaluation of the treatment effectiveness [12,13].

The obtained biomedical data were processed using the STATISTICA for Windows software system (version 13.0). The odds ratio (OR) was calculated with a 95% confidence interval (CI) for each suspected risk factor. The suspected risk factor was considered significant at OR > 1.0.

Verification of the diagnosis of COVID-19 was carried out according to the criteria presented in the “Prevention, diagnosis and treatment of new coronavirus infection (COVID-19)” temporary guidelines of the Ministry of Health of Russia. Confirmed COVID-19 was diagnosed in the presence of clinical symptoms in combination with typical lung CT scan features, and a positive test result for SARS-CoV-2 RNA using nucleic acid amplification methods.

Clinically significant neutropenia was defined as the number of neutrophils in peripheral blood <0.5 × 109/L, lymphocytopenia (the number of lymphocytes) <1.0 × 109/L.

Instrumental diagnostic methods included computed tomography (CT) of the chest in high resolution mode, and fibrobronchoscopy (FBS) with bronchoalveolar lavage (BAL) sampling. Tissue biopsy was performed according to indications.

Laboratory diagnosis of IA included microscopy and BAL and/or other respiratory biosubstrate cultures, galactomannan (GM) tests and histological examinations of biopsies. Samples of BAL were clarified in KOH 10% (diluted in 10% aqueous glycerin solution) with the addition of a fluorescent marker (calcofluor white). The presence of septated hyphae branching at an angle of 45° was noted in a fluorescent microscope. The material was cultured on a Saburo medium and incubated for 10 days at a temperature of 28 °C and +37 °C to allow the growth of fungi. The GM in blood serum and BAL was determined by enzyme immunoassay using a specific diagnostic test system—PLATELIA^®^ Aspergillus (BIO-RAD Laboratories, Des Plaines, IL, USA). According to the recommendations of ECMM/ISHAM, 2020, optical density indexes ≥1.0 in BAL or ≥0.5 in blood serum were considered significant [9]. Histological samples were prepared from the surgical, biopsy material, stained with hematoxylin-eosin, and the Gomori–Grocott method and PAS-reaction were implemented for fungal identification.

The PubMed and Web of Science databases, and www.aspergillus.org.uk (accessed on 10 October 2021), were analyzed using the following keywords ‘invasive aspergillosis’, ‘*Aspergillus* spp.’, ‘COVID-19’, ‘COVID-19-associated pulmonary aspergillosis’, ‘CAPA’.

## 3. Results

The study groups had similar demographic characteristics. The age of CAPA patients ranged from 34 to 82 years, the median age was 62 years. Male patients predominated at 69% (Table 1).

Despite the fact that background diseases did not significantly differ in both groups, our study showed that CAPA develops more often in patients with decompensated diabetes mellitus (DM) (29% vs. 7%, *p* = 0.0003), as well as in patients with active (out of remission) hematological and oncological diseases requiring antitumor therapy. CAPA developed more frequently in COPD patients (13% vs. 5.5%, *p* = 0.09).

Our study showed that the majority of patients of both groups (88% vs. 77%, *p* = 0.1) had lymphocytopenia <1.0 × 10^9^/L; however, the duration of lymphocytopenia significantly differed in CAPA and COVID-19 without IA, and the median was 15 (5–100) vs. 9 (2–42) days, respectively, *p* = 0.00002, (Table 2).

Herewith, prolonged (more than 10 days) lymphocytopenia was detected in 83% vs. 37% of patients, *p* = 0.006. Systemic glucocorticoids (GCS) were used to treat COVID-19 in most patients of both groups (88%), but CAPA developed more often at high doses of GCS (>60 mg/day of prednisone-equivalent dose) (46% vs. 16%, *p* = 0.01). Another significant risk factor was use of biological immunosuppressors (inhibitors of the IL-6 and IL-1ß receptors, such as tocilizumab, sarilumab and canakinumab) (42% vs. 20%, *p* = 0.01). Clinically significant neutropenia preceded the development of CAPA in 9% of cases (*p* = 00006).

The odds ratio (OR) was calculated to assess the impact of the identified risk factors on the development of IA. It was shown that the probability of IA developing in COVID-19 patients increased with lymphocytopenia for >10 days (OR = 8.156 (3.056–21.771), *p* = 0.001), decompensated DM (29% vs. 7%, (OR = 5.688 (1.991–16.246), *p* = 0.001), as well as the dose of GCS > 60 mg/day prednisone-equivalent (OR = 4.493 (1.896–10.647), *p* = 0.001) and the use of monoclonal antibodies targeting the IL-1ß and IL-6 receptors (OR = 2.880 (1.272–6.518), *p* = 0.01) (Table 3).

An analysis of the clinical data showed that CAPA was characterized by severe course. CAPA patients were in the ICU for a long time (median period was 15.5 vs. 6 days, *p* = 0.0004). Despite the fact that the majority of patients of both groups (62% vs. 60%, *p* = 0.7) had progressive respiratory failure that required oxygen support, CAPA patients more frequently needed mechanical ventilation (52% vs. 15%, *p* = 0.004), and acute respiratory distress syndrome (ARDS) developed in 31% vs. 18% of patents, *p* = 0.02. Another clinical feature of CAPA was hemoptysis (36% vs. 3%, *p* = 0.0001) (Table 4).

**Figure 1:**
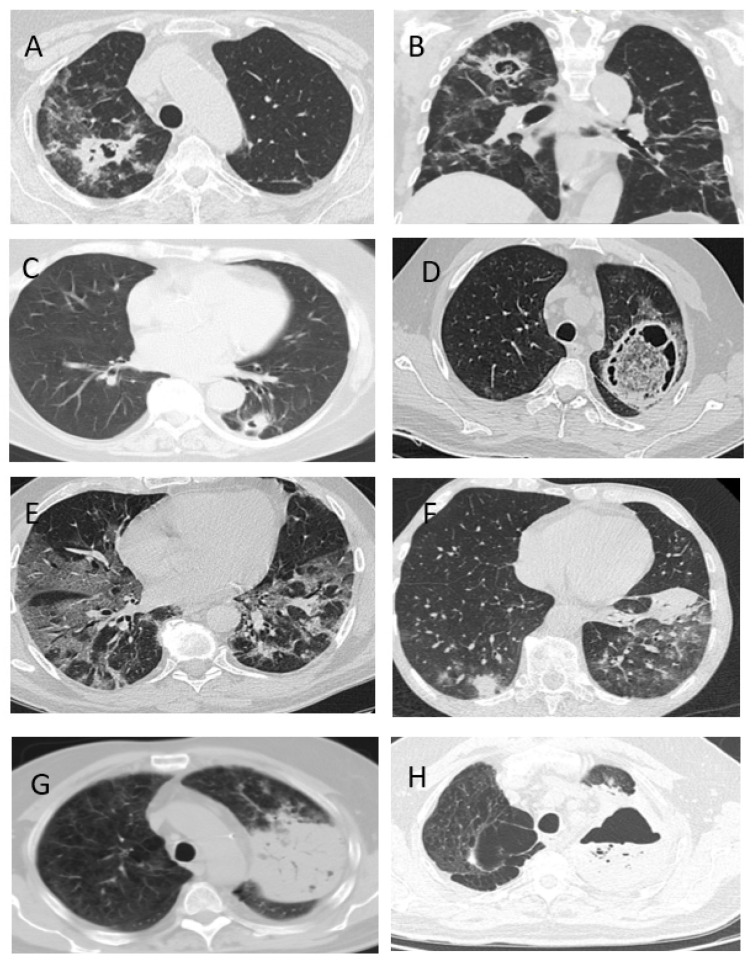
Chest CT scans of the patients with CAPA. (**A**,**B**) Patient S., 71 years, with CAPA and decompensated diabetes mellitus. There were cavities of destruction in the upper lobe of the right lung. (**C**) Patient G., 66 years, with CAPA and rheumatoid arthritis. There were infiltrations with areas of destruction (sickle sign) in S6 of the left lung. (**D**) Patient C, 35 years, with CAPA and diabetes mellitus with ketoacidosis. Large cavity with content localized in S1 + 2 of the left lung. (**E**) Patient K., 62 years, with CAPA and the debut of diabetes mellitus. There were multiple bilateral infiltrations with “frosted glass” sites and areas of consolidation. (**F**) Patient J., 68 years, with CAPA and multiple myeloma out of remission. On CT-scans in both lungs, there were polysegmental foci of “frosted glass” compaction and consolidation areas. (**G**,**H**) Patient C., 59 years, with CAPA. On CT-scans that showed increased dynamic infiltration and hydrothorax, destruction appeared in line with the level of fluid.

**Figure 2:**
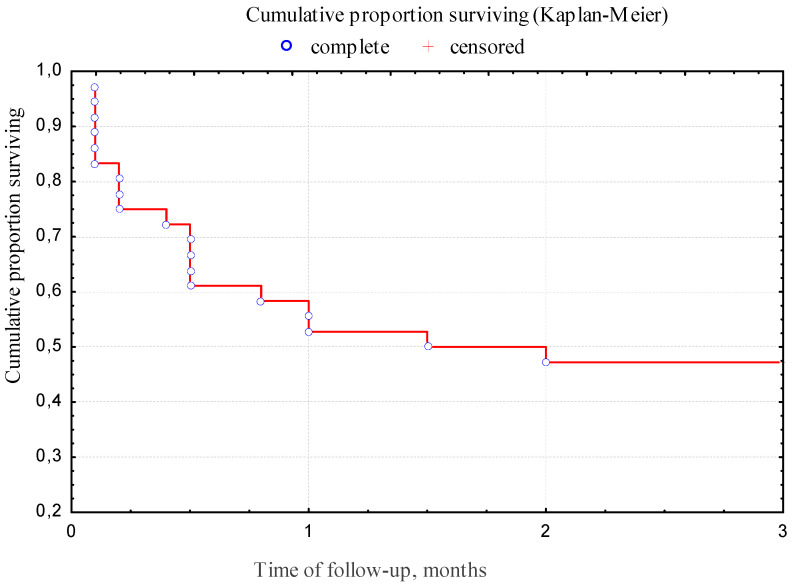
The overall 12-week survival of CAPA patients.

Lung lesion was typical for all CAPA patients and aspergillus tracheobronchitis was detected in 7% of patients. Infiltrations in the lung tissue were frequent radiologic signs of CAPA (89% vs. 59%, *p* = 0.004), and about a quarter of patients developed hydrothorax (26% vs. 11%, *p* = 0.03). The formation of destruction cavities was a distinctive feature of IA in this group of patients and was detected in 44% vs. 1% of subjects, *p* = 0.00001 (Figure 1 and Figure 2).

Laboratory diagnostics of IA included microscopy and culture of BAL (47%) and/or tracheobronchial aspirate (2%), GM testing in BAL (62%), and/or blood serum (9%), and biopsy histological examination (7%). Our study showed that the detection of GM in BAL in COVID-19 patients was the most valuable, and the GM test in serum was rarely positive (Table 5).

The mycelium specific for *Aspergillus* spp. was detected by microscopy in 24% of CAPA patients, and the growth of *Aspergillus* spp. in cultures from BAL was obtained in 31% of patients.

The causative agents of CAPA were *A. fumigatus* (44%), *A. niger* (31%) and *A. flavus* (6%), as well as unidentified *Aspergillus* spp. (19%); two or more *Aspergillus* spp. were isolated in 12% of cases.

All CAPA patients included in the study received antifungal therapy. The basic treatment was voriconazole as monotherapy or in combination with other antifungals in 91% of patients. Caspofungin was used in 27% of patients; amphotericin B (4%) and echinocandins (4%) were rarely used. Combinations of antifungals were used in 13% of patients. The duration of therapy ranged from 2 to 90 days (median—25.5). Surgical treatment was not performed.

The overall survival rate of CAPA patients for 12 weeks was low and amounted to 47.2% (Figure 2).

## 4. Discussion

Patients with severe COVID-19 had marked violations of local (epithelial damage, ciliary clearance, etc.) and systemic immunity (lymphocytopenia, CD4-cytopenia, etc.) due to the viral infection itself and the use of GCS and immunosuppressants, which can be accompanied by bacterial and fungal superinfections, including IA [14,15,16].

Most of the CAPA cases occurred in ICU patients with prolonged lymphocytopenia (83%), the use of GCS (88%), as well as biological immunosuppressants (42%). In 31% of patients, CAPA was associated with ARDS, and 42% of patients required mechanical ventilation. Published research results indicate a high incidence of CAPA in similar cohorts of patients: in the Netherlands (31 patients on ventilators, 19% CAPA) [4], France (27 patients on ventilators, 33% CAPA) [5], and Germany (19 patients on ventilators, 26% CAPA) [3]. According to the literature, the frequency of CAPA among all patients with COVID-19 varied from 0.1% to 9.7% [17]. Among patients with COVID-19 admitted to the ICU, the frequency of CAPA averaged 10.2% (varies from 1.0% to 39.1%), and on artificial ventilation, this increased to 19–47.4% [2,17,18,19,20,21]. Recent cohort studies showed the incidence of SARS in ICU patients being from 10 to 15%. In patients with any of the EORTC/MSGERC host factors, the CAPA frequency increased to 30% [22]. Prattes et al. also noted in their study that the incidence of CAPA varied in different centers from 1.7% to 26.8% (median—10.7%), while CAPA was significantly more common among elderly patients, invasively ventilated patients and patients receiving tocilizumab [23].

According to the data set from different studies, the risk factors of CAPA include age being over 62 years and body weight being over 80 kg, male gender, the use of GCS before and during treatment in the ICU, the use of immunosuppressive drugs (IL-1ß and IL-6 receptor inhibitors, etc.), prolonged (median—15 days) lymphocytopenia (<1.0 × 10^9^ L), neutropenia (<0.5 × 10^9^/L), decompensated DM, COPD, ARDS, long-term treatment in the ICU, prolonged artificial ventilation, malignant neoplasms, cytostatic or immunosuppressive therapy before the ICU and the use of extracorporeal membrane oxygenation (ECMO) [3,10,13,14,17,22,23,24,25].

In our study, we obtained similar results, which revealed the following statistically significant risk factors: the use of GCS ≥ 60 mg/day (OR 3.296 (1.438–7.555), *p* = 0.03), the use of IL-1ß inhibitors and IL-6 receptor (OR 3.083 (1.379–6.895), *p* = 0.01), lymphocytopenia (<1.0 × 109/L) for more than 10 days (OR 3.285 (1.556–6.938), *p* = 0.006) and decompensated DM (OR 5.688 (1.991–16.246)), *p* = 0.003). At the same time, CAPA occurred more often in men (69%), and the median age was 62 (34–82) years. Most of our patients (71%) were in the ICU for a long time (median—15.5 days), ARDS developed in a third of patients (31%), and ventilation support received by 42% of patients. Patients with CAPA were significantly more likely to have oncological and oncohematological diseases out of remission (24% vs. 2%, *p* = 0.03). Neutropenia was a rare risk factor (9%).

Lung damage frequently develops in CAPA cases, aspergillosis tracheobronchitis occurs less often, while the clinical signs are nonspecific: fever refractory to antibiotic therapy and increasing respiratory failure [10,13,26]. In our cohort, lung damage occurred in 100% of CAPA patients, and aspergillosis tracheobronchitis in 7%. We did not detect any cases of hematogenous dissemination with ≥2 organs involvement in our patients. Patients with CAPA had increases body temperature above 38.5 °C that were resistant to antibiotic drugs (98% vs. 85%, *p* = 0.007), cough (89% vs. 72%, *p* = 0.002), ARDS (31% vs. 18%, *p* = 0.02), hemoptysis (36% vs. 3%, *p* = 0.0001), and chest pain (24% vs. 9%, *p* = 0.05) significantly more often compared with the control group.

One of the main difficulties of the differential diagnosis of lung lesions in CAPA patients is the non-specificity of radiologic signs especially in the late stages of the disease. For example, changes typical for COVID-19 of the “frosted glass” type may be associated with acute lung damage as a result of the toxic effect of drugs, and may also be mistakenly interpreted as signs of pneumocystis pneumonia. Some atypical radiologic symptoms detected in COVID-19, in particular the presence of cavities, may indicate other infections, such as bacterial abscessed pneumonia [24]. Nevertheless, the appearance of cavitation in the lungs should require the exclusion of CAPA, since this sign is not typical for COVID-19. There is an additional difficulty in interpreting radiological manifestations in patients with severe COVID-19, since lung lesions in ARDS can mimic CAPA [13].

Our experience also suggests that radiologic signs of CAPA are non-specific. In most of our patients (93%), we detected bilateral lung lesions, mainly with infiltrations (89%). The destruction cavities were noted in 47% of patients, and hydrothorax in 26%.

In all patients, CAPA was diagnosed on the basis of clinical data, CT-scans of the lungs, results of microbiological examination of BAL, and detection of GM in BAL and/or blood serum. The results of our study indicate that the BAL samples are preferred for the diagnosis of aspergillosis. In our patients, the diagnosis of CAPA was confirmed by GM in BAL in 56% of patients, and by GM in serum in 7%. The low diagnostic value of the GM in serum was proven in several recent studies [10,13,17,24,26].

The main causative agent of CAPA in our patients was *A. fumigatus* (44%), which corresponds to the results of other studies [26].

Early antifungal therapy is mandatory for the successful treatment of CAPA. All our patients received antifungal therapy. Voriconazole was mainly used for CAPA treatment in 91% patients, and 13% of patients received combination therapy. The average treatment duration was 25.5 days. Surgical intervention was not performed. According to the ECMM/ISHAM 2020 recommendations, the drugs of choice for the treatment of CAPA are voriconazole and isavuconazole, and combination therapy can be used if the initial monotherapy is ineffective or if IA is combined with other invasive mycoses (mucormycosis, etc.) [13].

It was determined that IA leads to increased mortality of COVID-19 patients by 16–35% [10,24,27]. For example, a retrospective analysis of the clinical data of patients with CAPA from various countries for the period from 1 March to 31 August 2020 established a 50% mortality rate within 12 weeks [17]. In the cohort study provided by Janssen et al., it was also noted that mortality of patients with CAPA in the ICU was significantly higher (52%) than in patients without CAPA (34%) [22]. In the report of the working group of Verweij et al., mortality in patients with CAPA varied from 44% to 71.4% in different countries [26]. In the study of Bartoletti et al., a high 30-day mortality was also observed in patients with CAPA and ARDS compared to patients without IA (44% vs. 19%), while IA was a statistically significant independent risk factor for mortality in patients with COVID-19 in the ICU (odds ratio (OR), 3.53; 95% CI, 1.29–9.67; *p* = 0.014) (10). A later multicenter, multinational cohort study including 20 centers in nine countries verified that CAPA was an independent strong predictor of ICU mortality [23].

Our data correspond to the results of other researchers. The overall 12-week survival rate in our CAPA patients was 47.2%. One of the reasons for high mortality may be the low concentration of voriconazole in the plasma of CAPA patients; therefore, monitoring of the concentration of this drug in blood plasma is recommended [26]. According to Hatzl et al., antifungal prophylaxis was associated with a reduced risk of developing IA but did not affect survival in patients with COVID-19 in the ICU. Randomized controlled trials are needed to evaluate the efficacy and safety of primary antifungal prophylaxis in these patients [28].

## 5. Conclusions

1. CAPA develops mainly on the background of diabetes (33%), hematological or oncological diseases (31%), and COPD (13%).
2. The probability of CAPA significantly increases with lymphocytopenia for >10 days (OR = 8.156 (3.056–21.771), *p* = 0.001), decompensated diabetes (29% vs. 7%, (OR = 5.688 (1.991–16.246), *p* = 0.001), use of steroids at a prednisone-equivalent dose > 60 mg/day (OR = 4.493 (1.896–10.647), *p* = 0.001) and monoclonal antibodies to IL-1ß and IL-6 (OR = 2.880 (1.272–6.518), *p* = 0.01)
3. CAPA is characterized by pulmonary involvement (100%), and rarely characterized by trachea and bronchi (7%).
4. Severe disease course with prolonged (median—15.5 (5–60) days) stays in the ICU (71%), mechanical ventilation (52%) and ARDS (31%) are typical in CAPA patients.
5. The clinical signs of CAPA are nonspecific, but typically include: fever (98%), cough (89%) and hemoptysis (36%). The radiological signs of CAPA are foci of consolidation (89%) and destruction (47%), and hydrothorax (26%).
6. The most effective method of diagnosing CAPA is the GM test in BAL.
7. The overall 12-week survival rate of patients with CAPA was 47.2%.

## Figures and Tables

**Table 1:** Characteristics of the study and control groups.

	CAPA		COVID-19 without IA		*p*-Value
	n = 45	%	n = 90	%	
Demographics					
males	31	69%	60	67%	0.66
females	14	31%	30	33%	
age median (years)	34–82 62		25–82 63		0.67
background diseases					
hematological diseases	9	20%	11	12%	0.2
lymphomas acute leukemia MM CLL others	6 1 2 - -	13% 2% 4%	6 1 1 2 1	7% 1% 1% 1%	
oncology	5	11%	5	5.5%	0.1
active hematological/oncological disease	11	24%	2	2%	0.03
DM decompensated DM	15 13	33% 29%	17 6	19% 7%	0.06 0.0003
COPD	6	13%	5	5.5%	0.09
ARF/CRF	5	11%	8	9%	0.6

n—number of patients; MM—multiple myeloma; CLL—chronic lymphoid leukemia; DM—diabetes mellitus; COPD—chronic obstructive pulmonary disease; ARF/CRF—acute renal failure/chronic renal failure.

**Table 2:** CAPA risk factors.

	COVID-IA		COVID-19 without IA		*p*-Value
	n/N	%	n/N	%	
neutropenia <0.5 × 10^9^/L	4/45	9%	0/90		0.006
duration (min-max)/ Me (days)	5–25 10		-		
lymphocytopenia <1.0 × 10^9^/L	38/43	88%	68/88	77%	0.1
duration (min-max)/ Me (days)	5–100 15		2–42 9		0.00002
lymphocytopenia >10 days	29/35	83%	32/86	37%	0.006
glucocorticoids (GCS):	38/43	88%	77/88	88%	0.7
GCS >60 mg/d in prednisone-equivalent dose	17/37	46%	14/88	16%	0.01
inhibitors of receptors IL-1β and IL-6	18/43	42%	16/80	20%	0.01

n—number of patients with identified risk factor; N—total number of patients in the study group with available data.

**Table 3:** Analysis of CAPA risk factors.

Risk Factors	CAPA	COVID-19 without IA	OR (95% CI)	*p*-Value
	n/N(%)	n/N(%)		
decompensated DM	13/45 (29%)	6/90 (7%)	5.688 (1.991–16.246)	0.001
lymphocytopenia >10 days	29/35 (83%)	32/86 (37%)	8.156 (3.056–21.771)	0.0001
GCS >60 mg/d in prednisone-equivalent dose	17/37 (46%)	14/88 (16%)	4.493 (1.896–10.647)	0.001
inhibitors of receptors IL-1β and IL-6	18/43 (42%)	16/80 (20%)	2.880 (1.272–6.518)	0.01

n—number of patients with identified risk factor; N—total number of patients in the study group with available data.

**Table 4:** Clinical and radiological features of CAPA.

Features	CAPA n = 45		COVID-19 without IA n = 90		*p*-Value
N	%	n	%		
fever	44	98%	62/73	85%	0.007
cough	40	89%	42/53	72%	0.002
chest pain	10/42	24%	4/45	9%	0.05
respiratory failure 2-3-4 (requiring O_2_ or ventilation)	28	62%	54	60%	0.7
ARDS	14	31%	16	18%	0.02
hemoptysis	16	36%	3/87	3%	0.0001
ICU	32	71%	57	63%	0.4
total days in ICU Me	5–60 15.5		1–55 6		0.0004
mechanical ventilation	14	52%	8/54	15%	0.004
CT-signs					
bilateral lesion	42	93%	75/80	94%	0.8
infiltrations	40	89%	37/63	59%	0.004
the “frosted glass” symptom	33	73%	64/80	80%	0.3
destruction cavity	21	47%	1	1%	0.00001
the “halo” symptom	-	-	-	-	
hydrothorax	10/38	26%	10/88	11%	0.03

n—number of patients; ARDS—acute respiratory distress syndrome; ICU—intensive care unit.

**Table 5:** Laboratory diagnostics of CAPA.

Method	Result	
	n	%
microscopy (+)	11	24%
culture (+)	14	31%
GM in blood (+)	3	7%
GM in BAL (+)	25	56%
histology (+)	3	7%

n—number of positive results; %—the proportion of positive results from the total number of CAPA patients.

## Data Availability

Data are available in the Department of Clinical Mycology, Allergy and Immunology, Kashkin Research Institute of Medical Mycology North-Western State Medical University named after I.I.Mechnikov, Saint-Petersburg, Russia.
